# Asynchronous Distance Learning Performance and Knowledge Retention of the National Institutes of Health Stroke Scale Among Health Care Professionals Using Video or e-Learning: Web-based Randomized Controlled Trial

**DOI:** 10.2196/63136

**Published:** 2025-03-04

**Authors:** Avinash Koka, Loric Stuby, Emmanuel Carrera, Ahmed Gabr, Margaret O'Connor, Nathalie Missilier Peruzzo, Olivier Waeterloot, Friedrich Medlin, Fabien Rigolet, Thomas Schmutz, Patrik Michel, Thibaut Desmettre, Mélanie Suppan, Laurent Suppan

**Affiliations:** 1 Division of Emergency Medicine Department of Anaesthesiology, Clinical Pharmacology, Intensive Care and Emergency Medicine Geneva University Hospitals and Faculty of Medicine, University of Geneva Geneva Switzerland; 2 Genève TEAM Ambulances Emergency Medical Services Geneva Switzerland; 3 Stroke Centre Department of Clinical Neurosciences Geneva University Hospitals and Faculty of Medicine, University of Geneva Geneva Switzerland; 4 Department of Ageing and Therapeutics University of Limerick Hospital Group University Hospital Limerick Limerick Ireland; 5 Stroke Unit Division of Neurology Fribourg Cantonal Hospital Fribourg Switzerland; 6 Care training center Fribourg Cantonal Hospital Fribourg Switzerland; 7 Division of Emergency Medicine Fribourg Cantonal Hospital Fribourg Switzerland; 8 Stroke Centre Division of Neurology, Department of Clinical Neurosciences Lausanne University Hospital Lausanne Switzerland; 9 Division of Anesthesiology Department of Anaesthesiology, Clinical Pharmacology, Intensive Care and Emergency Medicine Geneva University Hospitals and Faculty of Medicine, University of Geneva Geneva Switzerland

**Keywords:** stroke, e-learning, video, medical education, randomized controlled trial, knowledge retention, knowledge acquisition, NIHSS, National Institutes of Health Stroke Scale, learner satisfaction

## Abstract

**Background:**

Stroke treatment has significantly improved over the last decades, but the complexity of stroke cases requires specialized care through dedicated teams with specific knowledge and training. The National Institutes of Health Stroke Scale (NIHSS), widely used to assess neurological deficits and make treatment decisions, is reliable but requires specific training and certification. The traditional didactic training method, based on a video, may not adequately address certain NIHSS intricacies nor engage health care professionals (HCPs) in continuous learning, leading to suboptimal proficiency. In the context of time-constrained clinical settings, highly interactive e-learning could be a promising alternative for NIHSS knowledge acquisition and retention.

**Objective:**

This study aimed to assess the efficacy of a highly interactive e-learning module compared with a traditional didactic video in improving NIHSS knowledge among previously trained HCPs. Furthermore, its impact on knowledge retention was also assessed.

**Methods:**

A prospective, multicentric, triple-blind, and web-based randomized controlled trial was conducted in 3 Swiss university hospitals, involving HCPs previously trained in NIHSS. Invitations were sent through email, and participants were randomized to either the e-learning or traditional didactic video group through a fully automated process upon self-registration on the website. A 50-question quiz was administered before and after exposure to the training method, and scores were compared to assess knowledge acquisition. The quiz was repeated after 1 month to evaluate retention. Subjective assessments of learning methods that is, user satisfaction, probability of recommendation, perceived difficulty, and perception of duration, were also collected through a Likert-scale questionnaire. A sample size of 72 participants were deemed necessary to have an 80% chance of detecting a difference of 2 points in the postcourse quiz between groups at the 5% significance level.

**Results:**

Invitations to participate were sent through email to an estimated 325 HCPs. 174 HCPs enrolled in the study, of which 97 completed the study course. Both learning methods significantly improved NIHSS knowledge, with an improvement of 3.2 (range 2.0-4.3) points in the e-learning group and of 2.1 (1.2-3.1) points in the video group. However, the e-learning group performed better, with higher scores in knowledge acquisition (median score 39.0, IQR 36.0-41.0 vs 37, IQR 34.0-39.0; *P*=.03) and in knowledge retention (mean score 38.2, 95% CI 36.7-39.7 vs 35.8, 95% CI 34.8-36.8; *P*=.007). Participants in the e-learning group were more likely to recommend the learning method (77% vs 49%, *P*=.02), while no significant difference was found for satisfaction (*P*=.17), perceived duration (*P*=.17), and difficulty (*P*=.32).

**Conclusions:**

A highly interactive e-learning module was found to be an effective asynchronous method for NIHSS knowledge acquisition and retention in previously NIHSS-trained HCPs, and may now be considered for inclusion in NIHSS training programs for HCPs.

**International Registered Report Identifier (IRRID):**

RR2-10.3390/healthcare9111460

## Introduction

### Background

Despite the decline in stroke incidence and mortality since 1990, the absolute number of stroke cases are increasing due to the steep growth in the global population, along with an aging demographic and a greater burden of risk factors across many parts of the world [1]. Hence, stroke remains a major public health issue due to its high morbidity rates [2]. Acute reperfusion therapies, such as intravenous thrombolysis and thrombectomy, improve functional and survival prognosis after stroke [3,4]. However, patients must be carefully screened and selected, and these procedures must be performed within a limited timeframe, due to risks and complications associated with the treatment [3].

The National Institutes of Health Stroke Scale (NIHSS) is widely used around the world to triage, select treatment strategies, monitor and follow-up stroke patients [3,5]. First described in the late 1980s, it gained popularity after the publication of the first successful acute stroke treatment study [5,6]. The reliability of the NIHSS has been demonstrated across diverse populations of health care professionals (HCPs) [7,8]. However, prior training and certification are required [9-14] as it is a complex scale with numerous subtleties. In Switzerland, national stroke center and stroke unit certification procedures specify that all personnel must be appropriately trained. In the French-speaking part of Switzerland, all HCPs working in stroke units should be familiar with NIHSS. However, the training program, usually comprised of formal teaching sessions with a senior neurologist and bedside clinical training, is non standardized and center-specific [15-17].

Constraints of working in these 24-hour acute care units, such as unexpected inflow of patients, and weekend and night shift patterns, can limit HCPs exposure to regular clinical training sessions. The absence of time constraints and the self-paced nature of asynchronous digital learning methods such as electronic learning (e-learning) modules have contributed to their popularity in medical education [18], and have been extensively used during the COVID-19 pandemic [19,20]. Hence, a highly interactive e-learning module was created to teach the NIHSS [21]. Its use has been shown to improve user satisfaction, knowledge acquisition as well as dissemination in NIHSS-naive populations, when compared with the traditional didactic video alone [22,23].

Despite widespread usage of the NIHSS, little literature exists on the actual performance of HCPs using it on a regular basis. Over time, with routine practice and bedside training, some key aspects of NIHSS scoring may be overlooked, improperly practiced, or forgotten. Therefore, we hypothesized that even previously trained HCPs may have suboptimal NIHSS knowledge and that a refresher course using an e-learning module could be more effective than the traditional didactic video at improving their NIHSS proficiency. Further, we also hypothesized that knowledge retention at 1 month would be higher after following this interactive module than after reviewing the standard didactic video.

Given the heterogeneity of training described above, all HCPs authorized by their employers to care for neurological patients on a regular basis were considered eligible to participate in this study.

### Objectives

Our primary objective was to assess whether NIHSS-trained HCPs would significantly improve their NIHSS knowledge after completing a highly interactive e-learning module than after following the traditional didactic video. The secondary objective was to determine whether following either training material allowed better retention of knowledge at one month.

## Methods

### Study Design and Setting

**Figure 1:**
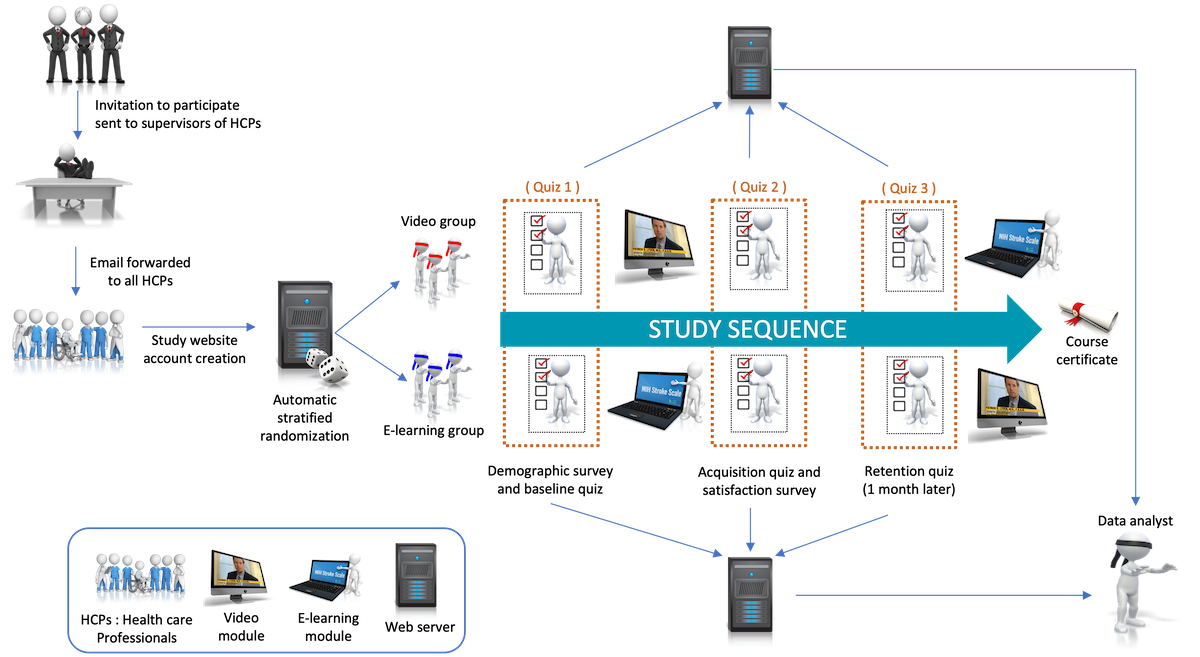
Study design.

This was a prospective, multicentric, web-based, triple-blind (participants, investigators, and data analyst) randomized controlled trial. Its main aim was to compare scores to a 50-question quiz, taken before and after exposure to the randomly assigned learning method, to assess the impact on knowledge acquisition. The same quiz was repeated 1 month later to assess the impact on knowledge retention. Figure 1 provides a detailed infographic of the study design.

This study was initially designed to include HCPs from the stroke units and neurology wards of 2 university hospitals in the French-speaking part of Switzerland, namely the Geneva University Hospitals and the Lausanne University Hospital. A third center, the Cantonal Hospital of Fribourg, joined the study a few weeks before its launch. Cantonal Hospital of Fribourg supervisors also extended the study invitation to all their personnel who potentially used the NIHSS on a regular basis, which included intensive care unit HCPs and emergency ward nurses.

The medical and nursing supervisors of all 3 centers forwarded an invitation email to their teams, containing a summary of the main points of the study and a link to the online platform. It is estimated that approximately 185 nurses and 140 physicians received this email. One author (L Suppan) sent weekly email updates to the supervisors of each center summarizing the number of participants in each hospital, stratified by profession. This email also detailed the number of participants at each stage of the study, thus enabling supervisors to monitor participation. Reminder emails regarding enrolment were left to the discretion of the supervisors. Automatic reminders were sent to participants who had registered on the platform but had not completed the required and appropriate study steps.

The initial invitation email was sent in July 2022. Enrollment and registration of new participants was disabled on March 31, 2023, and ongoing participants were invited to complete all steps by May 31, 2023. While the study platform remained accessible for scientific purposes such as further data extraction, users were no longer able to log in.

The study protocol has been previously published [24] and protocol deviations are reported in Multimedia Appendix 1.

CONSORT-EHEALTH (Consolidated Standards of Reporting Trials of Electronic and Mobile Health Applications and Online Telehealth) guidelines [25] and checklist (Multimedia Appendix 2) were followed, integrating relevant elements from the CHERRIES (Checklist for Reporting Results of Internet E-Survey) [26] and iCHECK-DH (Guidelines and Checklist for Reporting on Digital Health Implementations) guidelines [27].

The RCT was not prospectively registered because registration is not necessary for studies evaluating the effect of interventions on providers rather than patients [28].

#### Online Platform

The internet-based online study platform, compliant with General Data Protection Regulation, was created using the Joomla 3.10 content management system (Open Source Matters). Participants were assigned to specific user groups according to their progression using Joomla’s Access Control List. Custom PHP code was embedded using Sourcerer 9 (Regular Labs) to manage user group assignments.

The self-enrolment process was entirely automated. The first page of the website contained all necessary information regarding the study, and the consent information form was downloadable. Participants were explicitly informed that completing the registration process implied consent to participate in the study. Those who wished to enroll had to click on the logo of their hospital before acknowledging their profession (doctor or nurse) by clicking on a captioned image. This procedure invisibly randomized them either to the video or to the e-learning group using Gegabyte’s Random Article module 2.3. A 1:1 allocation was not possible due to the stratification by profession and center, and an unknown total number of participants. Participants were then asked to self-evaluate their current NIHSS knowledge (limited, moderate, or extended). The Membership Pro 3 component (Joomdonation) was then used to show a simple registration form that only asked for an email address and a secure password.

#### First Questionnaire

After completing the registration process, participants were asked to fill in a first questionnaire (Multimedia Appendix 3) designed to collect demographic data, administered using the Community Surveys 5.6 component (Shondalai) [29]. A detailed description of the first questionnaire can be found in the study protocol [24].

Participants then completed a 50-question quiz (Multimedia Appendix 4) designed to assess their baseline knowledge (quiz 1). Each question, worth 1 point, pertained to 1 of the 15 NIHSS items. All possible scores for that item were displayed and the participant had to choose the appropriate score. The quiz comprised a full NIHSS evaluation of 3 patients, for a total of 45 points. A total of 5 additional questions pertained to the general principles of NIHSS and certain subtleties, bringing the total to 50 points. All questions were mandatory, with multiple-choice answers to choose from.

Completion of this first quiz granted access to the allocated learning material as per randomization.

#### Learning Material and Second Questionnaire

The video group was given access to the original video created by Professor Lyden, of a duration of 53:07 minutes. The French subtitled version used for this study is freely available online under a Creative Commons license [30].

The e-learning group was given access to version 21c of the interactive e-learning module created under Storyline 3 (Articulate Global), also available freely online under a Creative Commons license [21]. This web-based module, which contains 184 slides, is divided into chapters following the NIHSS structure. Subtitled excerpts of Professor Lyden’s original video are embedded into the different chapters to illustrate clinical testing. This e-learning module has been extensively described in previous publications as well as in the protocol of this study [22-24].

Participants had full control of the allocated learning material, could go back and forth as often as needed, and pause, resume, restart, and review chapters, all without constraints or time limitations. Upon completion of the learning material, participants were then taken through the same 50-question quiz to assess knowledge acquisition (quiz 2).

This quiz was followed by a short satisfaction survey collecting subjective outcomes (ie, satisfaction, difficulty, duration, and likelihood of recommendation) using a 5-point Likert scale (Multimedia Appendix 5).

#### Knowledge Retention

Four weeks after completion of the satisfaction survey, participants were invited through email to complete the final quiz. They were asked to answer the 50-question quiz a third time to assess knowledge retention (quiz 3). At the end of this quiz, a certificate of participation was automatically generated that could be downloaded and printed. Only the email ID used for registration appeared automatically on the certificate, and participants were asked to write their names down after printing it. Each certificate was embedded with a unique barcode that could be scanned for verification of authenticity.

### Outcomes

The primary outcome was knowledge acquisition, assessed by the score on the second quiz, taken after completing the learning material.

Secondary outcomes were knowledge retention, assessed by the score on the third quiz undertaken at least 1 month after completion of the learning material, and subjective assessments of the followed learning method, namely user satisfaction, user perception of the duration, perceived difficulty, and the likelihood of recommending the learning material to a colleague.

### Participants and Sample Size

A total of 72 participants were required to have 80% chance of detecting a difference of 2 points in the postcourse 50-question quiz between groups at the 5% significance level. Considering a 40% attrition rate, we aimed to recruit 120 participants. A higher number of participants were accepted as participation did not entail any risk.

### Data Curation and Statistical Analysis

Data curation and statistical analysis were carried out using Stata (version 15.1; StataCorp). Data curation was done by 1 author (L Suppan), who assigned neutral names to the e-learning group and the video group. Another author (L Stuby) was then given this curated and blinded DTA file for data analysis. Continuous variables were first graphically described to look for the shape of the distribution. In case of doubtful normality, the Shapiro-Wilk test was applied. Then, depending on the variable’s distribution, either the Student *t* test or the Mann-Whitney *U* test were used. Results are reported either using mean (95% CI) or median (IQR).

A sensitivity analysis was performed by excluding those who had previously followed either the e-learning module, the original video from Patrick Lyden [20], or any other official NIHSS training. Categorical variables (eg, Likert scales) were first analyzed graphically, and then by Fisher exact test.

The time interval between the end of quiz 2 and the start of quiz 3 was also analyzed to ensure that the time elapsed before assessing retention was not lower than required as per the original protocol.

### Ethical Considerations

The regional ethics committee issued a “Declaration of no objection” in response to a jurisdictional query that we submitted (Req-2021-00543), as this study does not involve patients and falls outside the scope of the Swiss legislation regulating research on human subjects. Potential participants were provided with a detailed 4-page study information document, downloadable through a link on the study web page. Some of the key points noted in the document were that participation was on a voluntary basis, without any financial compensation, and data was fully anonymized before analysis. Furthermore, the document, and the study page, clearly indicated that registering for the study by creating an account was considered as acceptance to participate in the study. A screenshot of the study web page, and the consent information document, are annexed (Multimedia Appendices 6 and 7).

## Results

### Participants Characteristics

**Figure 2:**
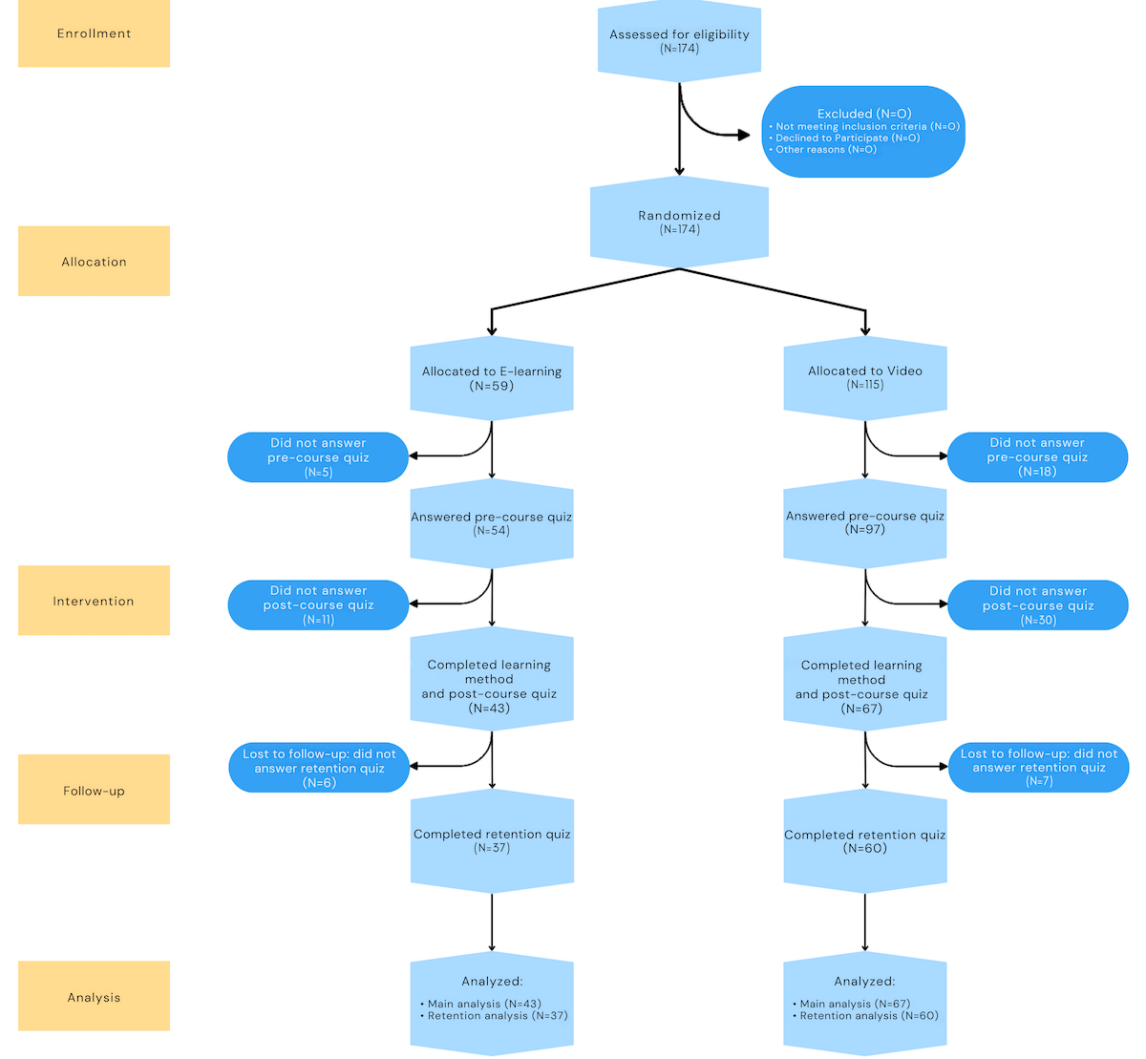
Study flowchart as per CONSORT guidelines. CONSORT: Consolidated Standards of Reporting Trials.

A total of 174 participants enrolled in the study. The fully automated randomization process attributed 59 participants to the e-learning group, and 115 participants to the video group (Figure 2). Characteristics of participants having completed the study path are described in Table 1. Given the high attrition rates, the characteristics of the participants who dropped out were also extracted and are displayed in Multimedia Appendix 8.

**Table 1:** Characteristics of participants having completed the study path.

Characteristics^a^				e-Learning group (n=43)		Video group (n=67)
Age (years), median (IQR)				32 (27-41)		32 (29-38)
**Gender, n (%)**						
		Female		33 (76.7)		52 (77.6)
		Male		10 (23.3)		15 (22.4)
**Profession, n (%)**						
		Nurse		26 (60.5)		49 (73.1)
		Physician		17 (39.5)		18 (26.9)
Time since certification (years), median (IQR)				6 (2-12)		8 (2-13)
**Center, n (%)**						
	HUG^b^		13 (30.2)		30 (44.8)	
	CHUV^c^		7 (16.3)		6 (9)	
	HFR^d^		23 (53.5)		31 (46.3)	
**Service, n (%)**						
	Ward		10 (23.3)		11 (16.4)	
	HDU^e^		13 (30.2)		34 (50.8)	
	ICU^f^		0 (0.0)		3 (4.5)	
	ED^g^		13 (30.2)		12 (17.9)	
	Other		7 (16.3)		7 (10.5)	
Time in main service (years), median (IQR)				2 (0-5)		3 (1-5)
**French mastery, n (%)**						
	None		0 (0)		0 (0)	
	Basic		0 (0)		0 (0)	
	Intermediate		1 (2.3)		0 (0)	
	Advanced		3 (7)		9 (13.4)	
	Proficient		39 (90.7)		58 (86.6)	
**English mastery, n (%)**						
	None		3 (7)		4 (6)	
	Basic		10 (23.3)		25 (37.3)	
	Intermediate		12 (27.9)		24 (35.8)	
	Advanced		17 (39.5)		14 (20.9)	
	Proficient		1 (2.3)		0 (0)	
**NIHSS^h^ training**						
	NIHSS internal training, n (%)		19 (44.2)		38 (56.7)	
	NIHSS official training, n (%)		2 (4.7)		5 (7.5)	
Using NIHSS since, (years), median (IQR)				3 (1-7)		3 (1-5)
**NIHSS use frequency, n (%)**						
	<1/month		6 (14)		3 (4.5)	
	1×/month		7 (16.3)		10 (14.9)	
	1×/week		8 (18.6)		14 (20.9)	
	1×/day		6 (14)		8 (11.9)	
	>1×/day		16 (37.2)		32 (47.8)	
**Comfort with NIHSS use, n (%)**						
	Not comfortable at all		3 (7)		1 (1.5)	
	Not so comfortable		7 (16.3)		6 (9)	
	Moderately comfortable		13 (30.2)		17 (25.4)	
	Quite comfortable		15 (34.9)		32 (47.8)	
	Very comfortable		5 (11.6)		11 (16.4)	
**NIHSS expertise, n (%)**						
	Limited		14 (32.6)		15 (22.4)	
	Moderate		20 (46.5)		22 (32.8)	
	Extended		9 (20.9)		30 (44.8)	
Baseline performance (quiz 1), mean (95% CI)				34.9 (33.7-36.2)		34.6 (33.8-35.3)

^a^Total may not be exactly 100% due to rounding.

^b^HUG: Geneva University Hospitals.

^c^CHUV: Lausanne University Hospital.

^d^HFR: Fribourg Cantonal Hospital.

^e^HDU: high dependency unit.

^f^ICU: intensive care unit.

^g^ED: emergency department.

^h^NIHSS: National Institutes of Health Stroke Scale.

### Participation According to Group Allocation

A difference of 10.5% in the participation rate was found, with 37/59 (62.7%) participants completing the study course in the e-learning group versus 60/115 (52.2%) in the video group.

### Knowledge Acquisition

Both learning methods had a positive impact on knowledge acquisition as both groups improved their scores. The improvement in the e-learning group was a mean of 3.2 points (range 2.0-4.3) and of 2.1 points (range 1.2-3.1) in the video group.

**Figure 3:**
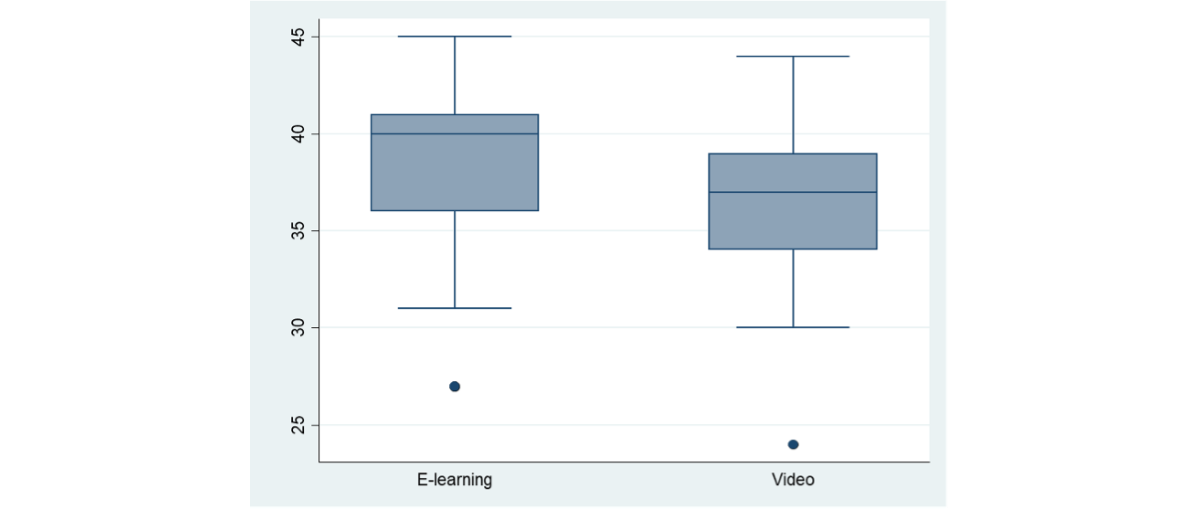
NIHSS knowledge acquisition scores (quiz 2) among groups. NIHSS: National Institutes of Health Stroke Scale.

Participants who followed the e-learning method performed better in the acquisition quiz than the participants in the video group (median score of 39, IQR 36-41 vs 37, IQR 34-39; *P*=.03; Figure 3).

### Knowledge Retention

**Figure 4:**
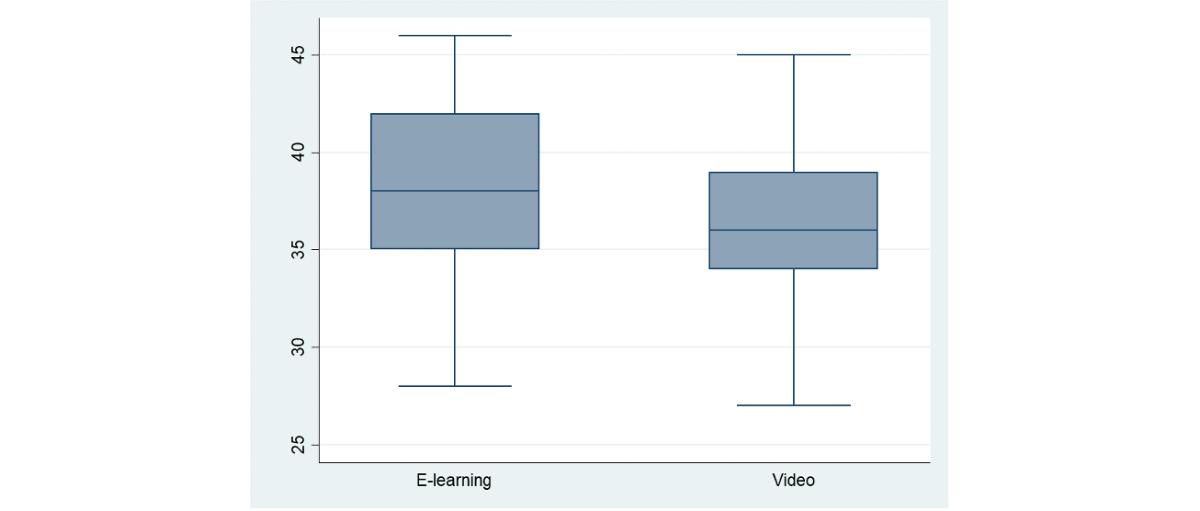
NIHSS knowledge retention scores (quiz 3) among groups. NIHSS: National Institutes of Health Stroke Scale.

The participants who followed the e-learning module had significantly higher scores on the retention quiz (38.2, 95% CI 36.7-39.7 vs 35.8, 95% CI 34.8-36.8; *P*=.007; Figure 4).

### Subjective Assessments

**Figure 5:**
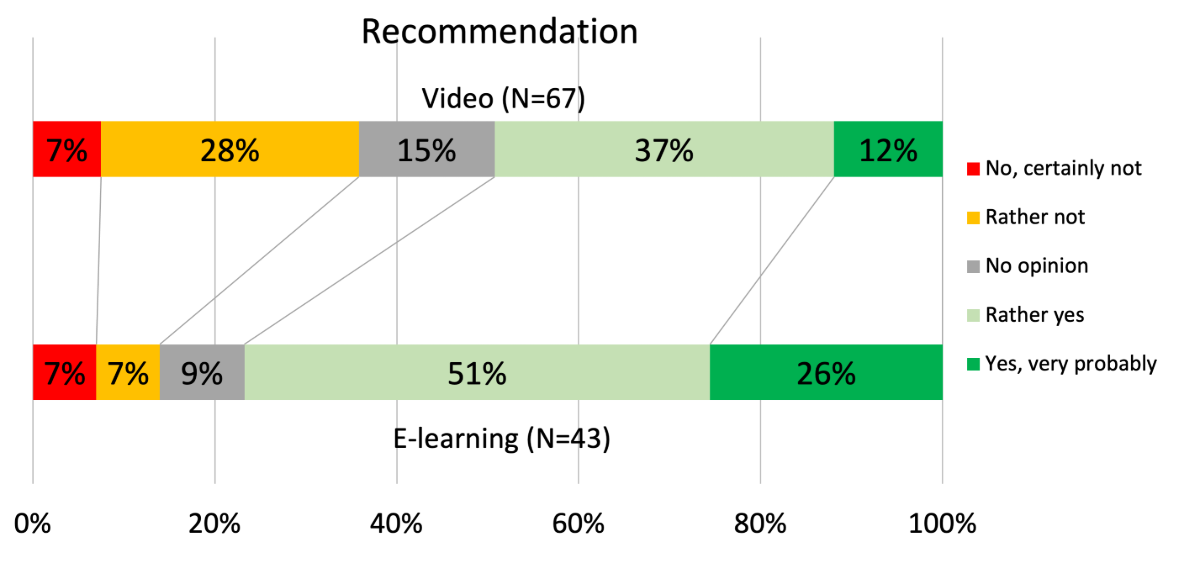
Likelihood of recommending the learning method to a colleague.

All participants who completed the learning method answered the subjective questionnaire. The likelihood of recommending the learning method was statistically higher in the e-learning group, with 33/43 (77%) participants in the e-learning group and 33/67 (49%) participants in the video group (*P*=.02; Figure 5). There was no significant difference regarding satisfaction (*P*=.17), perceived duration (*P*=.17), and difficulty (*P*=.32) (Multimedia Appendices 9-11).

### Sensitivity Analysis

The prespecified sensitivity analysis (ie, excluding those who had previously followed either learning method or any official NIHSS training) did not change the results (Table 2).

**Table 2:** Sensitivity analyses.

	e-Learning	Video	*P* value
Sensitivity analysis of primary outcome (knowledge acquisition), median (IQR), n	39 (37-41), 40	36 (34-39), 59	.02
Sensitivity analysis of knowledge retention, mean (95% CI), n	38.2 (36.6-39.7), 33	35.6 (34.5-36.7), 52	.007

### Time Interval Analysis

Time interval analysis confirmed that about a month had elapsed between the start of quiz 2 and the start of quiz 3 in both groups (e-learning versus video median of 36.0, IQR 27.4-49.0 versus 35.3, IQR 28.3-58.9 days).

## Discussion

### Principal Findings

This study shows that when compared with the traditional didactic video, a highly interactive e-learning module improves NIHSS knowledge acquisition and retention in HCPs caring for patients with stroke.

Decline in knowledge retention has been shown to be nonlinear, with a high percentage of decline measurable after only 4 weeks of acquisition [31]. The higher scores at quiz 3, undertaken at least 4 weeks after quiz 2, demonstrate that this interactive e-learning module also has a significant impact on knowledge retention.

Although both learning methods were considered equally difficult, participants of the e-learning group expressed a higher likelihood of recommending the learning method to colleagues, and thus would probably facilitate knowledge dissemination. Further, it is worth noting that the number of participants who completed the learning process was higher in the e-learning group by more than 10%, also hinting toward higher satisfaction in this group.

### Comparison to Previous Work

Previous studies have shown a positive impact of the e-learning module on knowledge acquisition among paramedics and medical students not familiar with the NIHSS [22,23]. However, this is the first study to show similar results in HCPs with prior NIHSS knowledge. This is also the first study to test for impact on knowledge retention as mentioned above.

### Strengths and Limitations

Several limitations must be acknowledged. First, attrition rates, as anticipated, were high in both groups. This is probably related to the fact that the entire study path takes a long time, requiring 4 to 5 hours to be completed and that there is currently a very high rate of fatigue among HCPs, with high turnover rates [32]. The study was conducted over a prolonged period of 11 months and participants were allowed to pause and resume the study at any time to try to minimize this issue.

Second, participants were informed that the study was to be completed individually and without any visual support such as a NIHSS form during the quiz. However, given the web-based asynchronous design of the study, we are unable to verify compliance to these points. The randomization however mitigates this issue, should it hold true.

Third, there is no certainty that multiple participants following different learning methods did not collaborate during this study, creating a potential contamination in the learning process. While a cluster-based randomization, with each center being an individual cluster, could have mitigated this risk, the large sample size required for such a design would have seriously impacted the feasibility of this study. In addition, based on their teaching experience with the study participants, the authors and supervisors strongly believe that the risk of such contamination is very low.

Fourth, the improvement of the score at each repetition could be partly induced by a priming effect. However, this effect would be similar across both groups and therefore should not induce any bias in data interpretation when comparing both groups.

Finally, trying to determine NIHSS knowledge by using a 50-question quiz cannot be considered fully representative of the skills in the clinical application of the score. However, this method has been used for the previous 2 decades to test NIHSS competency [11,14,33]. Its usage can therefore be considered valid to compare multiple learning methods.

This study also has some strong points, which are mentioned here. To our knowledge, this is the first paper that assesses the impact of an e-learning method on a population of previously trained HCPs, in terms of knowledge acquisition and retention. The protocol was published before starting this study, and deviations from the protocol have been thoroughly detailed in this paper (Multimedia Appendix 1). The randomization process was fully automated enabling us to guarantee the concealment of allocation, and data blinding was accomplished at the highest standard. The elapsed time between the second and third quizzes was assessed to ensure that the interval was indeed more than 1 month, thus ensuring compliance with the protocol.

Because language proficiency could have an impact on learning performance, participants were asked about their levels of English and French in the initial demographic questionnaire. Although randomization was intended to guarantee an equal balance of these elements in each group, the comparison allowed us to ascertain it.

The e-learning module itself also has certain advantages. Access to the official NIHSS training and certification could be limited by financial constraints and linguistic barriers, as most participating HCPs are French-speaking. This e-learning module is freely available online, its asynchronous learning design is particularly adapted to HCPs constraints, and it can easily be translated into different languages. The intrinsic design of the platform also enables quick updates of individual chapters if needed in the future. All these points allow easier dissemination of NIHSS knowledge and skills through e-learning than with the traditional didactic English video alone.

### Future Directions

Compared with previous studies in populations not familiar with the NIHSS [22,23], the overall scores are slightly higher. However, these scores are lower than could be expected given the specific study population of HCPs with previous NIHSS knowledge using the scale on a regular basis. Several hypotheses could explain this finding. First, baseline data analysis shows that 15% of participants declare themselves uncomfortable with the application of NIHSS, 26% consider themselves as having limited NIHSS knowledge, and only 7 participants have followed the official NIHSS training and certification. Furthermore, only about half the HCPs participating in this study declare having been through the internal training program of their institution. These low numbers could partly be explained by a high turnover currently seen in HCPs in general since the COVID-19 pandemic [34,35], with many new staff members who might have recently started working in these units as reflected by the low number of years the participants have been in their service. Moreover, some of the participants, such as Intensive care unit HCPs and emergency ward nurses, may have less exposure to NIHSS usage in their daily practice. Further studies should investigate the association between self-assessed level of NIHSS expertise, and frequency of exposition to the score, with the actual NIHSS knowledge.

The videos used for the quiz could also have an impact on the overall scores. A previous study [23], using the first edition of the e-learning module, had shown that the lack of video extracts, or graphic examples of neurological deficits, decreased the performance of candidates for certain chapters. An updated version integrating the missing video extracts showed that the performance of the same chapters improved [22]. However, feedback from previous study participants and discussions with experts correlate to say that some of the video excerpts in the e-learning or in the quiz could contain some ambiguity and leave room for some interpretation, for example, due to camera angles. We believe that this could in part explain some incorrect scoring and thus reduce the overall scores, and that replacing the videos with animated models demonstrating the neurological deficits more clearly could improve overall performance at testing.

### Conclusion

A highly interactive e-learning module was found to be an effective asynchronous method to improve NIHSS knowledge acquisition and retention, notably in previously NIHSS-trained HCPs. It may now be considered for inclusion in NIHSS training programs for HCPs.

## Appendix

Multimedia Appendix 1 Protocol deviations.

Multimedia Appendix 2 CONSORT-eHEALTH checklist (V 1.6.1).

Multimedia Appendix 3 First questionnaire.

Multimedia Appendix 4 50 Questions quiz.

Multimedia Appendix 5 Satisfaction Survey questionnaire.

Multimedia Appendix 6 Study webpage screenshot.

Multimedia Appendix 7 Study consent form.

Multimedia Appendix 8 Characteristics of all participants.

Multimedia Appendix 9 User satisfaction of learning method.

Multimedia Appendix 10 Perceived difficulty of the learning method.

Multimedia Appendix 11 Perceived duration of the learning method.

## Abbreviations

CHERRIES: Checklist for Reporting Results of Internet e-Surveys
CONSORT: Consolidated Standards of Reporting Trials
HCP: health care professional
iCHECK-DH: Guidelines and Checklist for Reporting on Digital Health Implementations
NIHSS: National Institutes of Health Stroke Scale

## Data Availability

The datasets generated during and/or analyzed during this study are available in the Yareta repository [36].
