# Supplementary material for: Asynchronous Distance Learning Performance and Knowledge Retention of the National Institutes of Health Stroke Scale Among Health Care Professionals Using Video or e-Learning: Web-based Randomized Controlled Trial
Source: J Med Internet Res. 2025 Mar 4;27:e63136. doi: 10.2196/63136 (PMC11920661; doi:10.2196/63136)
Supplement: Multimedia Appendix 1 [file jmir_v27i1e63136_app1.pdf]

Protocol deviations

Several protocol deviations must be mentioned:

- Clarification of the primary outcome: reviewing the published protocol before data analysis pointed out an inconsistency in defining the primary outcome. In the outcomes' section, we stated the primary outcome as the difference between the score on the 50-question quiz answered before and after following the allocated material. However, in the objectives, we stated that the primary objective was to determine if NIHSS knowledge improved more significantly in the e-learning group compared to the video; further, in the sample size calculation, we sought to look for a difference of 2 points in the post-course questionnaire. Prior to performing the statistical analysis, we clarified our primary outcome: as we did not expect to find a difference in baseline knowledge between both groups due to the randomized design of the study, and given that the main goal of this study was to evaluate the impact of the learning method on knowledge acquisition, the primary outcome was defined as knowledge acquisition, assessed by the score difference on the post-training quiz (quiz 2) between groups.
- Reporting of baseline knowledge: the initial protocol planned to provide a cross-sectional description of the baseline performance in addition to the current results. However, as a sizeable proportion of participants did not complete the study pathway but have answered the baseline quiz, the cross-sectional description will be reported in a separate paper, which will enable analysis of the complete dataset and enable more in-depth analysis (profession, self-assessed prior NIHSS experience, centre, self-assessed comfort with use, exposition to the scale, and gender). Baseline performance of those having completed the study pathway are mentioned in table 1.
- Statistical analysis: in the protocol, we had planned to analyse the data using linear regression models. However, considering that the randomized design of the study would also randomize

any confounding variables, we decided to simplify the statistical analysis before the end of data collection, as described in the "Data Curation and Statistical Analysis" section above.

- Time interval between assessment of knowledge acquisition and retention: The online platform was developed based on the assumption that participants would progress through all the components of each group, as detailed above, in a single session. Thus, the automatic email inviting participants to proceed to the third quiz was configured to be sent one month after activation of step 2, not after its completion. The authors quickly noticed that participants often paused their progression and resumed a few days or even weeks later. To manage this unforeseen issue, individual progression was reviewed on a weekly basis and the dispatch of the email inviting participants to access the retention questionnaire was adapted accordingly.
- Specific time interval outcomes: the voluntary pauses in progression described above make time interval comparison between groups uninterpretable. Before data compilation, it was decided to remove these outcomes from analysis and these variables were excluded from the compiled data set sent to the data analyst.
- Correction of affiliations: all centres provided continuing education credit (5 hours) for participation in the study. In this process, one staff supervisor noticed that the participation numbers provided by the authors did not tally with his list of participants. Data verification identified that some participants had clicked on the wrong hospital affiliation during the registration process. All email IDs were therefore screened and affiliations corrected whenever relevant. While this was straightforward when participants had used their professional email IDs, centre coordinators were asked to manually review specific addresses in case of doubt.
