# Supplementary material for: Asynchronous Distance Learning Performance and Knowledge Retention of the National Institutes of Health Stroke Scale Among Health Care Professionals Using Video or e-Learning: Web-based Randomized Controlled Trial
Source: J Med Internet Res. 2025 Mar 4;27:e63136. doi: 10.2196/63136 (PMC11920661; doi:10.2196/63136)
Supplement: Multimedia Appendix 3 [file jmir_v27i1e63136_app3.doc]

**Multimedia Appendix 1:** First Questionnaire

| **Page** | **Field** | **Original Question** | **English Translation** |
| --- | --- | --- | --- |
| 1 | Demographics | Age | Age ^a^ |
|  |  | Genre | Gender ^b^ |
|  |  | Années d’expérience clinique au total | Number of years of total clinical experience ^a^ |
|  |  | Années d’expérience clinique dans un service de neurologie et/ou de neurochirurgie | Number of years of clinical experience in a neurology and/or neurosurgery ward ^a^ |
|  |  | Maîtrise du Français | French proficiency ^c^ |
|  |  | Maîtrise de l’Anglais | English proficiency ^c^ |
|  |  | Dans quel service travaillez-vous principalement:   - Unité Cérébrovasculaire—surveillance continue - Unité Cérébrovasculaire-étage - Etage de neurologie - Soins intermédiaires de neurologie - Etage de neurochirurgie - Soins intermédiaires de   neurochirurgie   - Autre | In which ward do you most frequently work: ^b^   - Stroke unit-surveillance unit - Stroke unit-regular ward - Neurology ward - Neurology intermediate care unit - Neurosurgical ward - Neurosurgical intermediate care unit - Other |
| 2 | Prior NIHSS knowledge | Avez-vous effectué une formation interne du service pour apprendre à faire une évaluation NIHSS ? | Have you completed an in house NIHSS training? ^b^ |
|  |  | Avez-vous complété une formation certifiante officielle NIHSS ? | Have you completed the official NIHSS certification course? ^b^ |
|  |  | Nombre d’années de pratique avec l’échelle NIHSS | Number of years of clinical experience with the NIHSS scale ^a^ |
|  |  | Fréquence d’application du NIHSS:   - Plusieurs fois par jour - Environ 1 fois par jour - Environ 1 fois par semaine - Environ 1 fois par mois - Très rarement ou jamais | NIHSS use frequency: ^b^   - Many times per day - Circa once a day - Circa once a week - Circa once per month - Almost never or never |
|  |  | Je me sens à l’aise par rapport à l’application du NIHSS | I feel comfortable using the NIHSS ^c^ |

^a^ Regex: regular expression validation. ^b^ MCQ: multiple-choice question (only one answer accepted). ^c^ 5-point Likert scale.
