# Supplementary material for: Asynchronous Distance Learning Performance and Knowledge Retention of the National Institutes of Health Stroke Scale Among Health Care Professionals Using Video or e-Learning: Web-based Randomized Controlled Trial
Source: J Med Internet Res. 2025 Mar 4;27:e63136. doi: 10.2196/63136 (PMC11920661; doi:10.2196/63136)
Supplement: Multimedia Appendix 5 [file jmir_v27i1e63136_app5.doc]

**Multimedia Appendix 2:** Satisfaction Survey questionnaire

| **Page** | **Field** | **Original Question** | **English Translation** |
| --- | --- | --- | --- |
| Feedback regarding training | | Avez-vous déjà suivi cette méthode de formation au NIHSS ?   - Oui - Non | Have you previously completed this NIHSS learning method?   - Yes - No |
|  |  | Quand avez-vous suivi cette formation ?   - Il y a moins d’un mois - Il y a moins de 6mois - Il y a moins d’un an - Il y a plus d’un an - Je ne me souviens pas | When did you complete this training?   - Less than a month ago - Less than 6 months ago - Less than a year ago - More than a year ago - I don’t remember |
|  |  | Dans quel cadre avez-vous suivi cette formation ?   - Formation demandée par mon service - Trouvé cette formation sur internet par hasard - Information reçue par e-mail | In what context did you undergo this training?   - Required by my division - Found this training online by chance - Received information via email |
|  |  | Comment évaluez-vous le niveau de difficulté global de cette formation ?   - Très difficile - Difficile - Intermédiaire - Facile - Très facile | How do you evaluate the level of difficulty of this training?   - Very difficult - Difficult - Intermediate - Easy - Very Easy |
|  |  | Que pensez-vous de la durée de cette méthode de formation ?   - Trop longue - Un peu trop longue - Adéquate - Un peu trop courte - Trop courte | What do you think about the duration of this learning method?   - Too long - A little too long - Adequate - A little too short - Too short |
|  |  | Quel est votre niveau de satisfaction concernant la méthode de formation ?   - Pas du tout satisfait - Peu satisfait - Neutre / sans opinion - Satisfait - Très satisfait | What is your level of satisfaction regarding the learning method?   - Not at all satisfied - Not satisfied - Neutral / No opinion - Satisfied - Very satisfied |
|  |  | Recommanderiez-vous cette méthode de formation à vos collègues ?   - Non-certainement pas - Plutôt non - Pas d’opinion - Plutôt oui - Oui, très probablement | Would you recommend this learning method to your colleagues?   - Certainly not - Rather not - No opinion - Rather Yes - Certainly |
|  |  | Avez-vous des commentaires supplémentaires ?   - Texte libre | Do you have any other comments?   - Free text |
