# Supplementary material for: Asynchronous Distance Learning Performance and Knowledge Retention of the National Institutes of Health Stroke Scale Among Health Care Professionals Using Video or e-Learning: Web-based Randomized Controlled Trial
Source: J Med Internet Res. 2025 Mar 4;27:e63136. doi: 10.2196/63136 (PMC11920661; doi:10.2196/63136)
Supplement: Multimedia Appendix 6 [file jmir_v27i1e63136_app6.pdf]

cardiomobile.ch/nihss4/

NIHSS ACCUEIL

Etude/Formation NIHSS

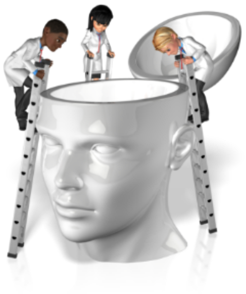

Il n'est désormais plus possible de s'inscrire sur la plateforme de l'étude NIHSS car le recrutement a pris fin au 31 mars 2023. Vous pouvez bien entendu vous y connecter si vous avez déjà un identifiant afin de terminer le parcours de formation ou pour récupérer votre certificat de formation.

Bonjour,

Nous vous remercions pour l'intérêt que vous portez à notre étude/formation sur le National Institutes of Health Stroke Score, ou NIHSS. Le but de notre étude est de déterminer si la méthode pédagogique proposée offre un rappel utile et efficace des connaissances nécessaires à l'application du NIHSS. Nous vous enverrons un courriel 1 mois après la fin de votre parcours afin de déterminer si la méthode pédagogique employée permet une bonne rétention des connaissances.

En vous connectant, vous consentez à participer à notre étude. Cliquez ici pour de plus amples informations.

Nous ne récoltons qu'un minimum d'informations à votre sujet, dont le but n'est en aucun cas de vous identifier. Vous pouvez à tout moment décider d'interrompre votre participation, sans avoir à vous en justifier. Vos responsables hiérarchiques n'auront jamais accès aux données spécifiques vous concernant. Il ne vous sera jamais demandé de décliner votre identité, et nous n'aurons besoin que d'une adresse e-mail pour pouvoir vous recontacter (et vous permettre de vous reconnecter sur ce site). Vous pouvez employer une adresse privée si vous le souhaitez.

Afin d'améliorer votre expérience de formation, vous devriez avoir activé le son sur votre appareil pour consulter le matériel de formation. L'emploi d'écouteurs est recommandé.

Les responsables de l'étude se tiennent à votre entière disposition pour toute question.

Adresse e-mail de contact: laurent.suppan@hcuge.ch

Bootstrap is a front-end framework of Twitter, Inc. Code licensed under MIT License.
Font Awesome font licensed under SIL OFL 1.1.
