# Supplementary material for: Asynchronous Distance Learning Performance and Knowledge Retention of the National Institutes of Health Stroke Scale Among Health Care Professionals Using Video or e-Learning: Web-based Randomized Controlled Trial
Source: J Med Internet Res. 2025 Mar 4;27:e63136. doi: 10.2196/63136 (PMC11920661; doi:10.2196/63136)
Supplement: Multimedia Appendix 7 [file jmir_v27i1e63136_app7.pdf]

IMPACT D'UN MODULE DE FORMATION À DISTANCE SUR LA CONNAISSANCE DE L'ÉCHELLE NIHSS (NATIONAL INSTITUTES OF HEALTH STROKE SCALE)

Cher.e collègue,

Nous vous proposons de participer à un projet de recherche, dont les détails sont explicités ci-dessous.

1. OBJECTIFS DU PROJET DE RECHERCHE

Le but de cette étude est de déterminer l'impact d'une méthode d'enseignement à distance sur les connaissances relatives à l'application de l'échelle NIHSS. Nous cherchons également à déterminer la rétention des connaissances dans ce contexte.

2. SÉLECTION DES PERSONNES POUVANT PARTICIPER AU PROJET

La participation est ouverte à tou.te.s les infirmier.e.s et tou.te.s les médecins internes/assistant.e.s des HUG et du CHUV ayant reçu une invitation par courrier électronique.

3. INFORMATIONS GÉNÉRALES SUR LE PROJET

Ce projet est effectué dans le respect des prescriptions de la législation helvétique. Les commissions cantonales d'éthique compétentes ont émis une déclaration de non-objection.

4. DÉROULEMENT POUR LES PARTICIPANTS

La page internet initiale offre des informations générales par rapport à l'étude et vous a permis d'accéder à ce document.

En cliquant sur le lien spécifique à votre institution, vous accédez à une page vous demandant de sélectionner votre profil professionnel et votre expérience par rapport au NIHSS. Après avoir cliqué sur votre sélection, un formulaire d'inscription en ligne vous est présenté. La seule information récoltée pouvant potentiellement permettre de vous identifier est votre adresse de courrier électronique. Vous êtes libre d'employer une adresse privée.

Les résultats aux différents questionnaires ne seront jamais mis à disposition en relation avec votre adresse de courrier électronique. Le responsable du traitement des données appartient à une unité de médecine d'urgence préhospitalière et ne vous connaît à priori pas personnellement. Il ne transmettra que les informations après les avoir dûment anonymisées. Vos responsables hiérarchiques n'auront jamais accès à vos scores personnels.

La participation à cette étude/formation se fait sur une base purement volontaire. Si vous ne désirez pas, pour une raison ou une autre, y participer, vous êtes parfaitement libre de ne pas le faire.

En créant un compte sur la plateforme informatique https://etude.nihss-study.ch, vous acceptez les conditions énoncées dans ce document et sur la page principale.

5. BÉNÉFICES POUR LES PARTICIPANTS

Votre participation au projet devrait vous permettre de mieux appréhender l'échelle NIHSS et de vous donner des indications utiles pour son application en clinique. Votre capacité à effectuer un examen neurologique de qualité dans un contexte d'AVC devrait s'en trouver renforcée.

6. DROITS DES PARTICIPANTS

Vous êtes libre d'accepter ou de refuser de participer au projet. Si vous choisissez de ne pas participer ou si vous choisissez de participer et revenez sur votre décision pendant le déroulement du projet, vous n'aurez pas à vous justifier. Vous pouvez à tout moment poser toutes les questions nécessaires au sujet de l'étude, auprès des investigateurs mentionnés à la fin de ce document.

7. OBLIGATIONS DES PARTICIPANTS

En tant que participant.e au projet, vous serez tenu.e de suivre attentivement la formation et de remplir les différents quiz.

8. RISQUES

En participant à ce projet, vous n'encourez aucun risque particulier.

9. CONFIDENTIALITÉ DES DONNÉES

Aucune donnée personnelle permettant de vous identifier ne sera récoltée hormis votre adresse de courrier électronique.

Dans le cas d'une publication, les données agrégées ne vous sont pas imputables en tant que personne. Votre nom n'apparaîtra jamais sur internet ou dans une publication. Tous les investigateurs et leurs auxiliaires sont tenus au secret professionnel. Toutes les directives relatives à la protection des données seront strictement respectées.

Il se peut que les données anonymes récoltées soient ultérieurement réutilisées à des fins d'analyse dans le cadre d'autres études scientifiques.

10. RÉMUNÉRATION DES PARTICIPANTS

Aucune rémunération ne sera octroyée pour votre participation.

Par votre participation, vous autorisez explicitement les investigateurs à utiliser les données anonymes obtenues à des fins de publication scientifique, et renoncez à toute requête de rétribution scientifique ou financière.

11. FINANCEMENT DU PROJET

L'étude est intégralement autofinancée par les investigateurs.

12. INTERLOCUTEUR(S)

Vous pouvez vous adresser à tout moment à l'un des interlocuteurs suivants :

Investigateurs principaux :

- Dr Laurent Suppan, 4 Rue Gabrielle-Perret-Gentil, Genève – laurent.suppan@hcuge.ch
- Dr Avinash Koka, 4 Rue Gabrielle-Perret-Gentil, Genève – avinash.koka@hcuge.ch

Co-investigateurs :

- Professeur Patrik Michel, Rue du Bugnon 21. CH-1011 Lausanne – patrik.michel@chuv.ch
- Dr Emmanuel Carrera, 4 Rue Gabrielle-Perret-Gentil, Genève – emmanuel.carrera@hcuge.ch
- M. Loric Stuby –18, Rue Docteur Alfred-Vincent, Genève – l.stuby@gt-ambulances.ch
- Dr Mélanie Suppan, 4 Rue Gabrielle-Perret-Gentil, Genève – melanie.suppan@hcuge.ch
