# Supplementary material for: Asynchronous Distance Learning Performance and Knowledge Retention of the National Institutes of Health Stroke Scale Among Health Care Professionals Using Video or e-Learning: Web-based Randomized Controlled Trial
Source: J Med Internet Res. 2025 Mar 4;27:e63136. doi: 10.2196/63136 (PMC11920661; doi:10.2196/63136)
Supplement: Multimedia Appendix 8 [file jmir_v27i1e63136_app8.doc]

**Multimedia Appendix 3:** Characteristics of all participants

|  | **E-learning (n=43)** | **Video (n=67)** | **Randomized to e-learning but did not complete study course (n= 16)** | **Randomized to video but did not complete study course (n= 48)** |
| --- | --- | --- | --- | --- |
| Age, years, median [quartiles]  - Missing data, n | 32 [27;41] | 32 [29;38] | 34 [28;40]  1 | 32 [30;39]  6 |
| Gender, n (%)  - Female  - Male  - Missing data, n | 33 (76.7)  10 (23.3) | 52 (77.6)  15 (22.4) | 11 (68.8)  4 (25.0)  1 (6.3) | 29 (60.4)  13 (27.1)  6 (12.5) |
| Profession, n (%)  - Nurse  - Physician | 26 (60.5)  17 (39.5) | 49 (73.1)  18 (26.9) | 9 (56.3)  7 (43.8) | 35 (72.9)  13 (27.1) |
| Time since certification, years, median [quartiles]  - Missing data, n | 6 [2;12] | 8 [2;13] | 8 [2;15]  1 | 7 [3;14]  6 |
| Center, n (%)  - HUG  - CHUV  - HFR | 13 (30.2)  7 (16.3)  23 (53.5) | 30 (44.8)  6 (9.0)  31 (46.3) | 6 (37.5)  3 (18.8)  7 (43.8) | 17 (35.4)  9 (18.8)  22 (45.8) |
| Service, n (%)  - Ward  - HDU  - ICU  - ED  - Other  - Missing data | 10 (23.3)  13 (30.2)  0 (0.0)  13 (30.2)  7 (16.3) | 11 (16.4)  34 (50.8)  3 (4.5)  12 (17.9)  7 (10.5) | 2 (12.5)  6 (37.5)  0 (0.0)  7 (43.8)  0 (0.0)  1 (6.3) | 13 (27.1)  11 (22.9)  2 (4.2)  13 (27.1)  3 (6.3)  6 (12.5) |
| Time in main service, years, median [quartiles]  - Missing data | 2 [0;5] | 3 [1;5] | 3 [0;6] .  1 | 3 [1;6] .  6 |
| French mastery, n (%)  - None  - Basic  - Intermediate  - Advanced  - Proficient  - Missing data | 0 (0.0)  0 (0.0)  1 (2.3)  3 (7.0)  39 (90.7) | 0 (0.0)  0 (0.0)  0 (0.0)  9 (13.4)  58 (86.6) | 0 (0.0)  0 (0.0)  1 (6.3)  3 (18.8)  11 (68.8)  1 (6.3) | 0 (0.0)  0 (0.0)  1 (2.1)  3 (6.3)  38 (79.2)  6 (12.5) |
| English mastery, n (%)  - None  - Basic  - Intermediate  - Advanced  - Proficient  - Missing data | 3 (7.0)  10 (23.3)  12 (27.9)  17 (39.5)  1 (2.3) | 4 (6.0)  25 (37.3)  24 (35.8)  14 (20.9)  0 (0.0) | 0 (0.0)  3 (18.8)  5 (31.3)  6 (37.5)  1 (6.3)  1 (6.3) | 0 (0.0)  15 (31.2)  19 (39.6)  4 (8.3)  4 (8.3)  6 (12.5) |
| NIHSS internal training, n (%)  - Missing data | 19 (44.2) | 38 (56.7) | 9 (56.3) .  1 (6.3) | 11 (22.9) .  6 (12.5) |
| NIHSS official training, n (%)  - Missing data | 2 (4.7) | 5 (7.5) | 1 (6.3)  1 (6.3) | 4 (8.3)  6 (12.5) |
| Time since NIHSS use, years, median [quartiles]  - Missing data, n | 3 [1;7] | 3 [1;5] | 4 [2;6] .  1 | 3 [1;6] .  6 |
| NIHSS use frequency, n (%)  - <1 / month  - 1x / month  - 1x / week  - 1x / day  - >1x / day  - Missing data | 6 (14.0)  7 (16.3)  8 (18.6)  6 (14.0)  16 (37.2) | 3 (4.5)  10 (14.9)  14 (20.9)  8 (11.9)  32 (47.8) | 1 (6.3)  1 (6.3)  2 (12.5)  2 (12.5)  9 (56.3)  1 (6.3) | 5 (10.4)  4 (8.3)  6 (12.5)  11 (22.9)  16 (33.3)  6 (12.5) |
| Comfort with NIHSS use, n (%)  - Not comfortable at all  - Not so comfortable  - Moderately comfortable  - Quite comfortable  - Very comfortable  - Missing data | .  3 (7.0)  7 (16.3)  13 (30.2)  15 (34.9)  5 (11.6) | .  1 (1.5)  6 (9.0)  17 (25.4)  32 (47.8)  11 (16.4) | .  2 (12.5)  2 (12.5)  3 (18.8)  7 (43.8)  1 (6.3)  1 (6.3) | .  3 (6.3)  5 (10.4)  14 (29.2)  18 (37.5)  2 (4.2)  6 (12.5) |
| NIHSS expertise, n (%)  - Limited  - Moderate  - Extended | 14 (32.6)  20 (46.5)  9 (20.9) | 15 (22.4)  22 (32.8)  30 (44.8) | 3 (18.8)  5 (31.3)  8 (50.0) | 10 (20.8)  21 (43.8)  17 (35.4) |

*Total may not be exactly 100% due to rounding*
