# Supplementary figures and images for: Asynchronous Distance Learning Performance and Knowledge Retention of the National Institutes of Health Stroke Scale Among Health Care Professionals Using Video or e-Learning: Web-based Randomized Controlled Trial
Source: J Med Internet Res. 2025 Mar 4;27:e63136. doi: 10.2196/63136 (PMC11920661; doi:10.2196/63136)

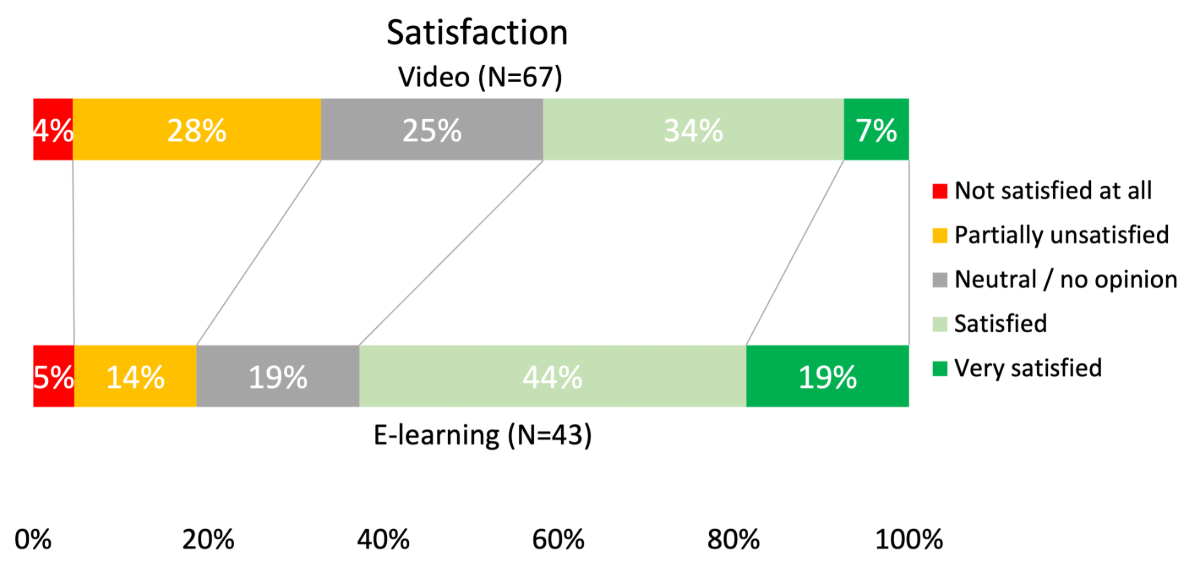

Supplement: Multimedia Appendix 9 [file jmir_v27i1e63136_app9.png]

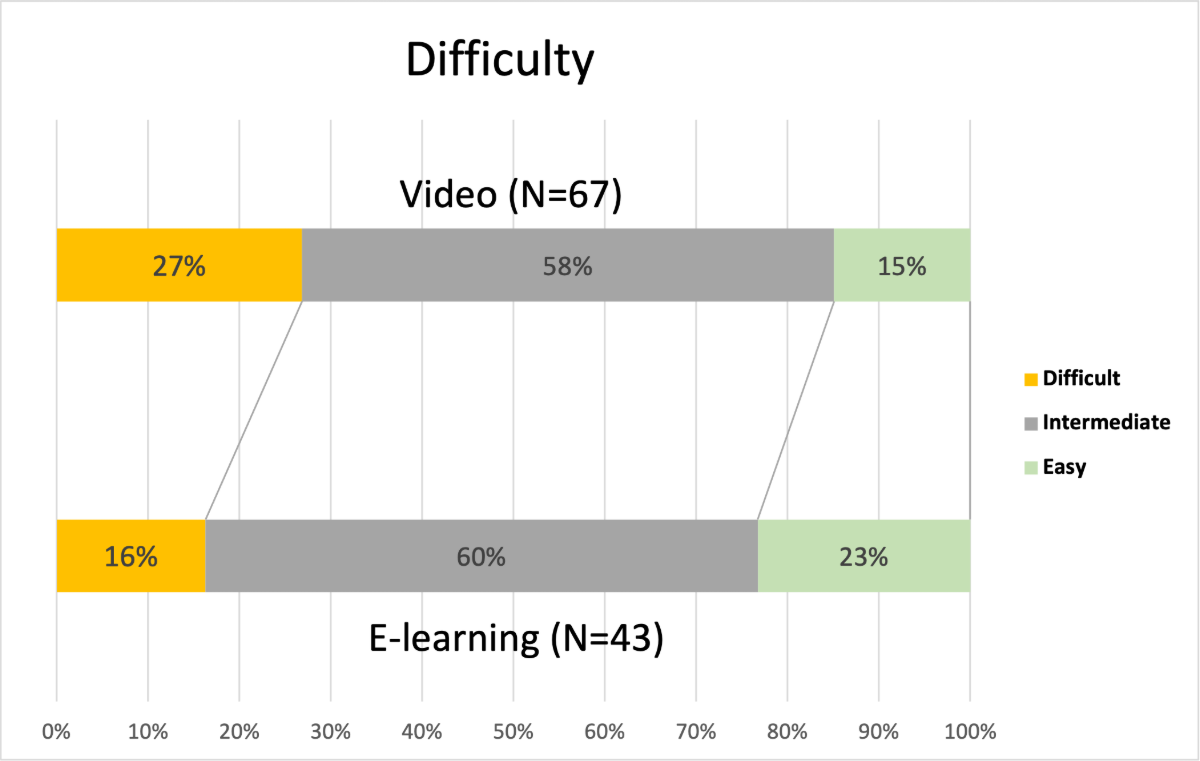

Supplement: Multimedia Appendix 10 [file jmir_v27i1e63136_app10.png]

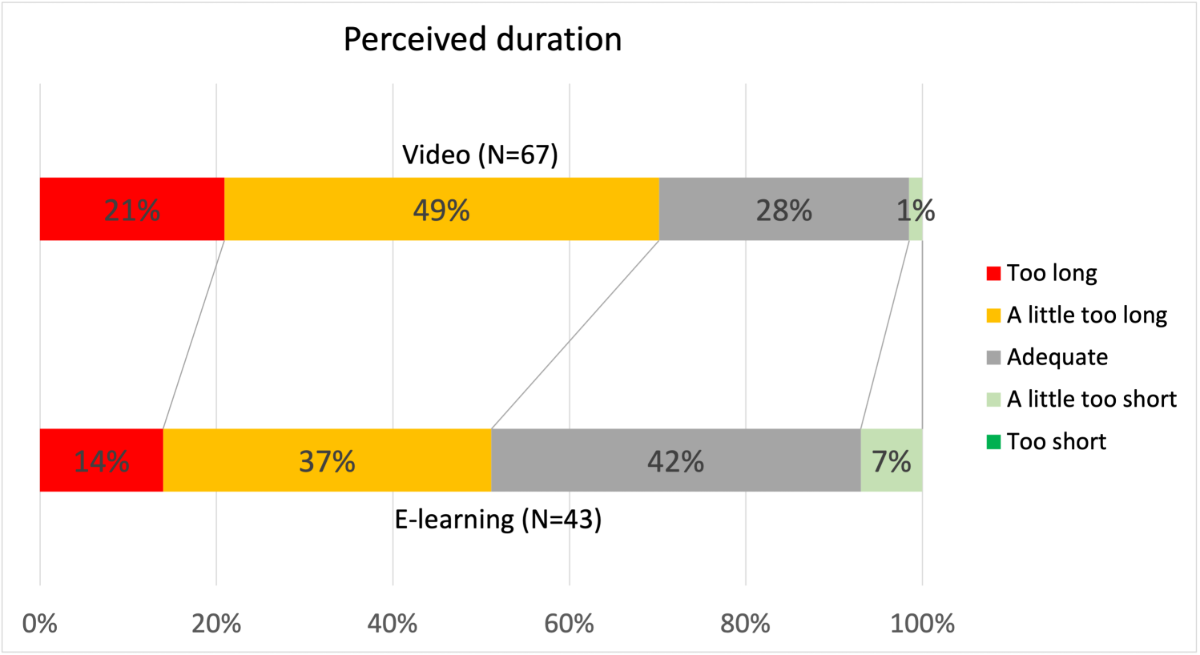

Supplement: Multimedia Appendix 11 [file jmir_v27i1e63136_app11.png]
